# Tako-tsubo cardiomyopathy after administration of ergometrine following elective caesarean delivery: a case report

**DOI:** 10.1186/1752-1947-4-280

**Published:** 2010-08-20

**Authors:** Abdulgazi Keskin, Ralph Winkler, Bernd Mark, Andreas Kilkowski, Timm Bauer, Oliver Koeth, Selcan Camci, Bernd Cornelius, Günther Layer, Uwe Zeymer, Ralf Zahn

**Affiliations:** 1 Department of Cardiology (Herzzentrum Ludwigshafen), Hospital Ludwigshafen, Academic Teaching Hospital of Johannes-Gutenberg-University of Mainz, Ludwigshafen am Rhein, Germany

## Abstract

**Introduction:**

Tako-tsubo cardiomyopathy (stress-induced cardiomyopathy or transient left ventricular ballooning) is characterized by clinical suspicion of an acute myocardial infarction with transient apical or midventricular dyskinesia of the left ventricle without significant coronary stenosis on angiography. The etiology of this disease remains obscure. One of the possible causes is myocardial ischemia induced by coronary vasospasm due to sympathetic activation. It has been hypothesized that the application of ergometrine could induce tako-tsubo cardiomyopathy.

**Case presentation:**

We report the case of a 28-year-old Turkish woman who developed tako-tsubo cardiomyopathy after administration of ergometrine for release of placenta and prevention of bleeding during the post-partum phase in the course of an elective caesarean delivery. Tako-tsubo cardiomyopathy was diagnosed by echocardiography and urgent cardiac magnetic resonance imaging. A coronary angiography was not performed because of the absence of myocardial necrosis or ischemia and signs of myocarditis on cardiac magnetic resonance imaging.

**Conclusion:**

This life-threatening disease should be excluded in the differential diagnosis by comparing the symptoms with those of typical heart failure, particularly after use of ergometrine.

## Introduction

Since the first description in 1991 by Dote *et al*.,[1], an increasing number of reports of tako-tsubo cardiomyopathy (CMP) have been published. This disease is typically seen in postmenopausal women aged from 58 to 77 years [2]. It is also present in about 1.2% of cases of troponin-positive acute coronary syndrome, with an atypical (midventricular) pattern found in 40% of those cases with tako-tsubo cardiomyopathy (1.2%). Intrahospital mortality is nearly 1%, and a 30-day mortality rate of 8.6% was reported in one study by Kurowski *et al. *[2].

## Case presentation

A 28-year-old healthy Turkish woman (height 166 cm, weight 75 kg), without any medical history was admitted to a peripheral hospital at 37 weeks gestation for an elective caesarean delivery. During the course of the delivery, intravenous short-term infusion of 0.2 mg methylergometrine and 30IE oxytocin was administered for easy release of the placenta and prevention of bleeding during the post-partum phase. There were no complications during delivery. Approximately 30 minutes after delivery, the patient developed severe distress and chest pain. On physical examination, rales were detected in both lungs (Killip class II). The patient was transferred to our hospital for further investigation.

On electrocardiogram, a sinus tachycardia (100/min) without ST-segment changes was seen. The patient's blood pressure was 100/60 mmHg and her pO_2 _was 52 mmHg without oxygen supplementation. Chest x-ray revealed severe fluid consolidation (N-terminal prohormone brain natriuretic peptide-brain natriuretic peptide value 3900 pg/ml). Oxygen and loop diuretics rapidly improved the patient's respiratory status. The initial two-dimensional echocardiography showed moderately reduced systolic left ventricular function with a midventricular hypokinesia. Left ventricular end diastolic diameter was normal. The ejection fraction as measured by the Simpson's method was 38%. Laboratory investigations found raised levels of troponin T (0.19 ng/ml,; normal < 0.03 ng/mL) and creatine kinase (356 U/L; normal < 145 U/L). The patient was started on diuretics and angiotensin-converting enzyme inhibitors, after which she recovered quickly and showed no respiratory distress or other signs of heart failure.

In the absence of any cardiovascular risk factors and the age of the patient, we decided against using coronary angiography for initial anatomic. We conducted contrast-enhanced cardiac magnetic resonance (CMR) imaging, which showed a circular midventricular hypokinesia and no delayed enhancement after gadolinium application. Neither myocardial necrosis nor ischemia were seen, therefore coronary angiography was not performed

The patient's cardiac enzymes normalized within three days after admission. Two-dimensional echocardiography showed that systolic left ventricular function had completely recovered without any wall motion abnormalities within those three days.

Based on the patient's history with absence of cardiovascular risk factors, mild cardiac enzyme elevation and CMR findings of midventricular hypokinesia without necrosis and ischemia, she was diagnosed with tako-tsubo CMP. Seven days after admission, the patient and her healthy newborn child were discharged.

## Discussion

Since the first description in 1991 by Dote *et al. *[1], an increasing number of reports of tako-tsubo CMP have been published. The condition is typically seen in postmenopausal women in the range from 58 to 77 years [2-4]. It is present in about 1.2% of cases of troponin-positive acute coronary syndrome, with an atypical (midventricular) pattern being found in 40% of those cases. It is suggested that the atypical version is a variation of typical tako-tsubo CMP produced by early recovery of function at the apex with apical ballooning [5]. Intrahospital mortality is nearly 1%, and a 30-day mortality rate of 8.6% was described in one study by Kurowski *et al. *[2].

Our case report is an atypical presentation of a midventricular tako-tsubo CMP in a 28-year-old woman occurring within 30 minutes after use of ergometrine in a caesarean delivery.

**Figure 1:**
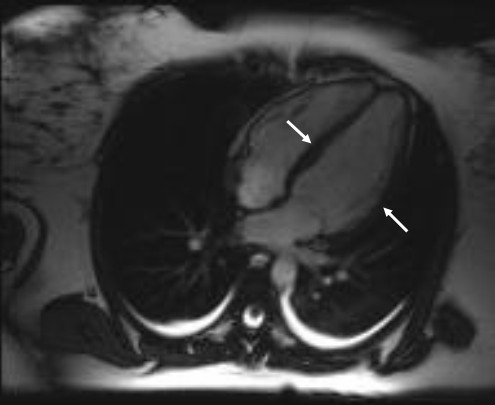
**Ventriculography by diastole with hypokinesia of midventricular segment (marked with white arrow)**.

**Figure 2:**
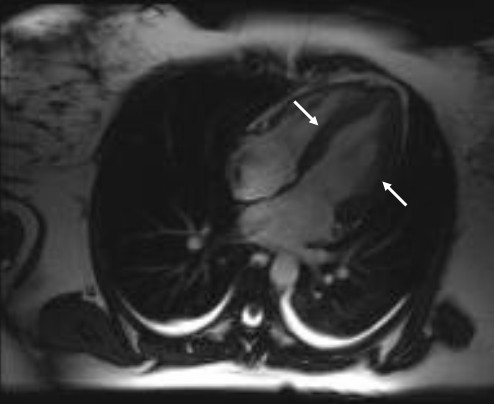
**Ventriculography by systole with hypokinesia of midventricular segment (marked with white arrow)**.

The suggested mechanism for tako-tsubo CMP is myocardial ischemia induced by vascular spasm due to sympathetic over-activation by a stressful situation [6,7]. A number of substances are known to induce vasospasm, and as shown by this report, ergometrine may also cause a tako-tsubo CMP. Ergometrine is a part of the ergot family of alkaloids, and is used for treatment of acute migraine attacks, to induce childbirth, and as in our case, to prevent post-partum haemorrhage. Ergometrine possesses structural similarity to several neurotransmitters, and has biological activity as a vasoconstrictor. These effects have been shown in both animal models and in human studies [8-10]. In the largest study, Akasaka e*t al*. reported 26 patients with angiographically documented normal coronary arteries and Prinzmetal's angina; the authors observed significant coronary vasospasm after ergometrine administration in all cases [10]. In our case, a combination of ergometrine administration and an extraordinary stress situation was present, so that the definite cause could not be isolated. Using CMR, dyskinesia of the left ventricle extending beyond the vascular bed of a single coronary artery and no delayed gadolinium enhancement were seen (Figure 1, Figure 2). A myocardial infarction was excluded by absence of necrosis and ischemia.

To the best of our knowledge, our case represents the first published report of a woman with tako-tsubo CMP after use of ergometrine in the course of caesarean delivery. In the literature, we found only one other case report of tako-tsubo CMP after ergometrine application, but this was in a 42-year-old woman with a hematologic disease and arterial hypertension [11].

As part of the differential diagnosis, we considered peripartum cardiomyopathy (PPCM), a rare, life-threatening disease of late pregnancy and the early postpartum period. However, this disease is typically seen in women with the following risk factors: age greater than 30 years,, multiparity, multiple pregnancies, African American ethnicity, obesity, and arterial hypertension. Hypokinesia of the left ventricle in PPCM is diffuse rather than segmental, and the left ventricular end-diastolic diameter is increased [12]. Our patient did not match any of these criteria, and she recovered left ventricular function rapidly; this is much slower PPCM than in tako-tsubo CMP [13].

## Conclusion

Tako-tsubo CMP should be considered in the differential diagnosis for patients with symptoms of acute heart failure particularly after use of ergometrine by caesarean delivery.

## Abbreviations

CMP: cardiomyopathy; CMR: cardiac magnetic resonance; ECG: electrocardiography; PPCM: peripartum cardiomyopathy

## Competing interests

The authors declare that they have no competing interests.

## Authors' contributions

AK was the assistant cardiologist who diagnosed the problem. RW and SC collected the data and helped draft the manuscript. BC performed the cardiac magnetic resonance. TB was a major contributor in writing the manuscript. All authors read and approved the final manuscript.

## Consent

Written informed consent was obtained from the patient for publication of this case report and any accompanying images. A copy of the written consent is available for review by the Editor-in-Chief of this journal.
